# Weekly Variations of Short-Duration Maximal Jumping Performance in Soccer Players: Exploring Relationships With Accumulated Training Load and Match Demands

**DOI:** 10.3389/fphys.2021.690353

**Published:** 2021-08-19

**Authors:** Rui Silva, Filipe Manuel Clemente, Francisco Tomás González-Fernández, André Bernardo, Luca Paolo Ardigò

**Affiliations:** ^1^Escola Superior Desporto e Lazer, Instituto Politécnico de Viana Do Castelo, Viana Do Castelo, Portugal; ^2^Instituto de Telecomunicações, Delegação da Covilhã, Lisboa, Portugal; ^3^Department of Physical Activity and Sport Sciences, Pontifical University of Comillas, Centro de Estudios Superiores Alberta Giménez, Palma, Spain; ^4^SER (Salud, Educación y Rendimiento) Research Group, Pontifical University of Comillas, Centro de Estudios Superiores Alberta Giménez, Palma, Spain; ^5^Department of Neurosciences, Biomedicine and Movement Sciences, School of Exercise and Sport Science, University of Verona, Verona, Italy

**Keywords:** football, readiness, muscle fatigue, athletic performance, sports training

## Abstract

**Purpose:** The aim of this study was 2-fold: (1) to analyze variations of short-duration maximal jumping performance in players exposed to a match and those who were not and (2) to analyze the relationships between changes in the short-duration maximal jumping performance and different accumulated training load and match demands measures.

**Methods:** Twenty-four professional soccer players (age: 20.3 ± 1.7 years) were monitored daily for their training load and match demands over 6 weeks. In addition, they performed a weekly short-duration maximal jumping performance test (72 h after the last match).

**Results:** Negative moderate correlations were found between percentage of change of countermovement jump (CMJ) height and Acummulated training load (ATL) of total distance (TD), high metabolic load (HML), accelerations (ACC), and decelerations (DEC) (*r* = −0.38, *p* = 0.004; *r* = −0.33, *p* = 0.013; *r* = −0.39, *p* = 0.003; and *r* = −0.30, *p* = 0.026). No correlations were found for match load (ML). TD, HML, ACC, and DCC (*r* = 0.27, *r* = 0.25, *r* = 0.31, and *r* = 0.22, respectively) were used to predict the percentage of change of CMJ height.

**Conclusion:** Match participation has negative effects on CMJ performance. The ATL of HML, ACC, DCC, and TD have a significant influence on both CMJ measures changes. Also, the ATL values of those metrics are the best predictors of the percentage changes of CMJ performance.

## Introduction

Research done over the past few years shows that modern soccer players experience substantial increases in high-intensity activities during training and competitions (Barnes et al., 2014). These increased demands are accompanied by a training schedule that limits recovery after a training week that ends with a highly demanding match. Increased weekly training loads, combined with demanding soccer matches, result in accumulated fatigue, which may negatively impact the performance of players during and after competitions and during subsequent training cycles (Haddad et al., 2013; Brownstein et al., 2017).

During a soccer match, athletes are expected to manifest temporary neuromuscular fatigue and performance reduction during different stages of the match, especially after short periods of high-intensity actions during both halves and near the end of the match (Bangsbo et al., 2007). Thus, after a single soccer match, athletes experience decreased physical performance for up to 72 h, as a result of fatigue (Silva et al., 2018). Indeed, a recent systematic review on this topic revealed that jump performance is impaired 72 h post-match while sprinting performance seems to recover at this time (Silva et al., 2018). Neuromuscular fatigue results in decreased force-production capacity concurrent with impairments in the muscle-stretch-shortening cycle (SSC), which are relevant issues for injury prevention and soccer performance maintenance perspectives (Debenham et al., 2015). Furthermore, regarding the recovery process after training and competition, the high between-subjects and between-weeks variations that might be present in a soccer team must be considered (Nédélec et al., 2012). In turn, over a repeated-sprint-ability exercise viz. a proxy for soccer running, a countermovement jump (CMJ)-based recovery was effective in increasing training load [e.g., the subjective rate of perceived exertion (RPE); Foster et al., 2001] without harming running mechanics (i.e., SSC).

Therefore, analyzing vertical jump performance variations after a soccer match is of paramount importance, especially if there is no other option available to assess neuromuscular fatigue after strenuous activity. In that sense, a recent study analyzed whether CMJ variables could track acute fatigue effects 24 and 48 h after a simulated soccer match. The results revealed that none of the CMJ metrics manifested any changes (Lombard et al., 2020). Indeed, it was previously shown that CMJ height did not vary during an in-season training week (without considering the match) and no correlations were found between weekly training loads and CMJ changes (Malone et al., 2015). On the contrary, another study documented that CMJ performance was negatively affected by training load and that there was a cumulative effect of weekly training loads on neuromuscular fatigue (Tavares et al., 2018). Interestingly, a recent systematic review analyzed the correlations between match external-load activities and acute (up to 24 h) and residual (up to 72 h) fatigue markers. The findings indicate that very high-intensity running activities (>5.5 ms^−1^) are strongly correlated with decreased neuromuscular performance based on the CMJ test, but only when they were assessed during the first 24 h after a match (Hader et al., 2019).

Also of great importance is understanding and monitoring neuromuscular fatigue of teams after a training week ending with a soccer match. Different assessment approaches and their related limitations have been documented (Wehbe et al., 2015; Carling et al., 2018; Troester and Duffield, 2019). By these means, coaches are equipped with enhanced knowledge of how their athletes respond to training and competitions, thereby allowing them to adjust training accordingly to prevent injuries and overtraining (Foster, 1998; McLean et al., 2010). From the different methodological assessments available for measuring the readiness of an athlete to train (i.e., detecting neuromuscular fatigue), the CMJ is a practical tool for detecting weekly neuromuscular performance changes (Claudino et al., 2017). Indeed, CMJ data analysis, when used to objectively quantify neuromuscular fatigue, revealed a lower coefficient of variation (<5%) for jump height than other relevant variables, thus confirming its reliability (Gathercole et al., 2015).

A short-duration maximal jumping testing protocol consisting of four CMJ repetitions interspersed by 3–5 s of recovery revealed that peak jump velocity might be more sensitive to fatigue detection when practitioners do not have any other means of assessing readiness to train than jump testing (Mathieu et al.,  2017). Indeed, practitioners can use this tool to monitor the effects of fatigue after team sports training and competitions as well as to assess resistance training neuromuscular readiness (Watkins et al., 2017).

Although many studies have investigated the effects of match loads on acute and residual neuromuscular fatigue (Silva et al., 2018), few studies have explored the relationships between match and accumulated loads with changes in neuromuscular performance (Rowell et al., 2018; Clemente et al., 2019). In addition, to the best of our knowledge, no study has investigated the associations between accumulated external training loads and changes in neuromuscular performance assessed *via* CMJ measures. For those reasons, and considering the importance of insights into the effects of weekly training loads and match demands on residual neuromuscular fatigue, the aims of this study were (1) to analyze variations in short-duration maximal jumping performance in players exposed to matches and those who were not and (2) to analyze the relationships between changes in the short-duration maximal jumping performance and different measures of accumulated training load and match demands.

## Methods

### Experimental Approach to the Problem

This study followed an observational analytic cohort design. The included players were analyzed over six non-consecutive weeks between October 13, 2020, and December 5, 2020 (i.e., from the beginning of the in-season period to the midseason) (Table 1).

**Table 1:** Characterization of the analyzed weeks in this study.

**Variable**	**Week 10–11**	**Week 11–12**	**Week 12–13**	**Week15–16**	**Week 16–17**
Month	October	October	November	November	December
Training sessions (*n*)	5 + M + 5	5 + M + 4	4 + M + 5	4 + M + 5	5 + M + 4
PPM (*n*)	16	14	No match	13	12
PNPM (*n*)	2	4	No match	2	2

*M, match; PPM, players participating in the match; PNPM, players not participating in the match*.

**Figure 1:**
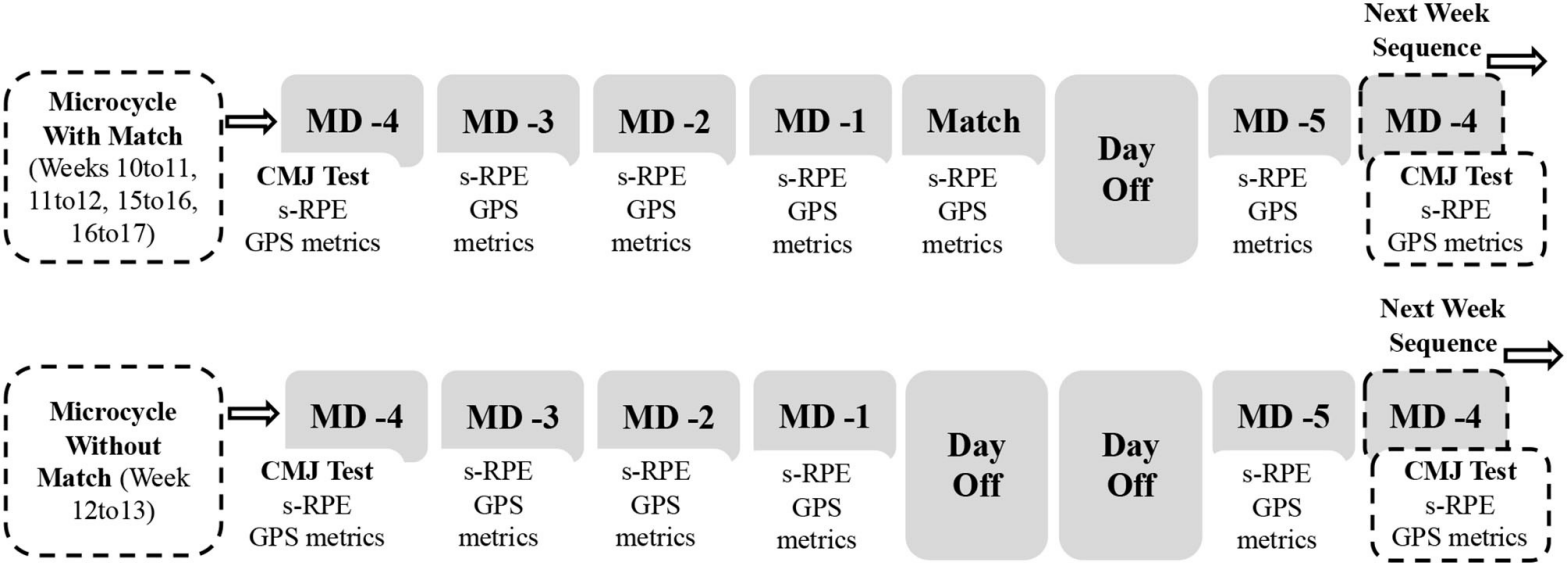
Representation of a weekly data collection sequence.

Eligible players were always tested 72 h after the most recent match using a short-duration maximal jumping performance test. Between one assessment and the next (i.e., the next week), the players were monitored daily by using internal and external load measures (Figure 1). Accumulated training load (ATL, sum of weekly training sessions loads) and match load (ML, average of in-between match loads) demands were registered. For the players not participating in the match, only the accumulated training load was considered.

### Participants

Twenty-four professional soccer players (age: 20.3 ± 1.7 years; body mass: 70.6 ± 7.0 kg; height: 179.1 ± 7.1 cm; years of experience: 11.5 ± 2.7; baseline CMJ height: 39.3 ± 5.2) voluntarily participated in this study. The players belonged to the same club, which competed in the third league of Portugal.

During each week of the observational period, the team had five training sessions (with an average session duration of 78.8 ± 15.6 min) and one official match. Only regular weeks (i.e., those with only one official match) were considered for analysis. For assessing readiness-to-train status of the teams, a short-duration maximal jumping performance test was conducted before the start of each match-day-4 (MD-4) training session. The training microcycle comprised of MD-5 (recovery), MD-4 (tension), MD-3 (duration), MD-2 (speed), and MD-1 (reactive speed).

For each observed week, the inclusion criteria for a player to be included in the data treatment were as follows: (1) the player participated in the pre- or post-match assessment (i.e., maximal jumping performance test), (2) the player was neither injured nor ill, nor did they miss any training sessions between the pre- and post-match assessments; (3) the player was not injured or ill the week before the assessment.

Before the beginning of the study, the players were informed about the study design and protocol as well as the risks and benefits of participating. After voluntarily deciding to participate, they signed an informed consent that explicitly stated that they were free to end the process at any time with no consequences. The study protocol was approved by a scientific council of the local university and followed the ethical standards of the Declaration of Helsinki for the study of humans.

### Short-Duration Maximal Jumping Performance Test

For the assessment of the short-duration maximal jump performance test, each player was requested to complete four CMJ repetitions interspersed by 3–5 s of recovery between jumps (Mathieu et al.,  2017). Players were instructed to maintain both hands on their hips during the four trials to keep their back straight without swinging their legs forward during the flight phase and to try to land in the same place they took off from, landing with the tips of their toes first. Participants were allowed to perform the CMJ at the depth that was most suitable for them.

All jumps were assessed using an Optojump photoelectric cells system (Microgate, Bolzano, Italy) connected to a portable computer with its respective software (Optojump software, version 1.10.19). Jump height was estimated as 9.8 × flight time^2^/8 and used for further analysis (Glatthorn et al., 2011). All measurements were conducted during the morning and before practice sessions. Although better jump performance was previously documented during the late afternoon, it seems that similar performance might be observed during the morning if certain criteria are met (Mirizio et al., 2020).

A standardized warm-up consisting of ankle mobility and knee range of motion exercises followed by three to five submaximal CMJs was conducted. After the warm-up, athletes rested for ~3 min before performing the four CMJ trials.

### Internal Load

For measuring the internal loads, RPE data were collected ~10–30 min after each training session, similarly to previous research (Foster et al., 2001). The CR-10 scale was used to quantify session effort of each player (Borg, 1998). Based on the CR-10 scale, 1 means “very light activity” and 10 means “maximal exertion.” Each player rated the effort individually, without being influenced by the other players, by using a smartphone questionnaire that asked, “How hard was your training?” All the players were previously familiarized with this kind of perceptual effort rating. The internal training loads were then obtained by multiplying the training session of each player, RPE (s-RPE), by the absolute time of each session (in minutes) (Foster, 1998).

### External Load

Each player used the same 18 Hz GPS unit (STATSports, Apex, Northern Ireland) during the period of the study. The GPS units have an integrated 100 Hz gyroscope, 100 Hz tri-axial accelerometer, and 10 Hz magnetometer. The GPS model used in this study was previously tested for its validity and reliability, revealing good levels of accuracy and variability at different speed thresholds and excellent interunit reliability for peak velocity (Beato et al., 2018; Beato and de Keijzer, 2019). The GPS units were placed in a specific vest in which the unit was fixed between the scapulae. The data collected during training sessions and matches were imported and processed in the STATSport Sonra software (version 3.0).

The following measures were collected daily during each training session and match: (1) total distance (TD: consisting in the total distance covered by players); (2) distance covered at high-speed running (HSR: distances covered at a speed of 19.8 km h^−1^ or above); (3) high metabolic load distances (HML: distances covered at a speed of >19.8 km h^−1^ while accelerating/decelerating at ≥2 m s^−2^); (4) high-intensity accelerations and decelerations (ACC and DEC: number of accelerations and decelerations at ≥3 m s^−2^); (5) sprint distances at 80% of the maximal speed (_80%_SD: distances covered at a speed corresponding to 80% of the actual maximal speed of each player).

### Statistical Procedures

For the treatment of the data, we use adequate statistical methods to calculate percentages and central and dispersion parameters (arithmetic mean and SD). The normal distribution of data was tested using the Kolmogorov–Smirnov test. CMJ pre-match and CMJ post-match measurements were assessed using a two-way, mixed-design ANOVA for group condition (played or not played match in-between) and time condition (pre- or post-match). Posteriorly, planned comparisons were performed to evaluate differences between times. A repeated-measure ANOVA was used to analyze the CMJ height (pre- and post-match) and times (week 10–11, 11–12, 12–13, 15–16, and 16–17). Effect size is indicated with Cohen's *d* for *t*-tests [0.2 (small); 0.5 (medium) and >0.8 (large)] and partial eta squared for Fs. A Pearson's correlation coefficient *r* was used to examine the relationship between the percentage of change of CMJ [CMJ (pre-match – post-match)] and the remaining variables (TD, HSR, HML, Sprint, ACC, DCC, CR-10, and sRPE). To interpret the magnitude of these correlations, we adopted the following criteria (Granier et al., 1995): *r* ≤ 0.1, trivial; 0.1 < *r* ≤ 0.3, small; 0.3 < *r* ≤ 0.5, moderate; 0.5 < *r* ≤ 0.7, large; 0.7 < *r* ≤ 0.9, very large; and *r* > 0.9, almost perfect. Regression analysis was used to model the prediction of both percentages of change from remaining variables with positive correlation. Data were analyzed by using the software Statistica (version 10.0; Statsoft, Inc., Tulsa, OK, USA). For all analyses, significance was accepted at *p* < 0.05.

## Results

Descriptive statistics were calculated for each variable (refer to Tables 2, 3, for more information).

**Table 2:** Descriptive statistics (mean ± SD) for the pre- and post-match countermovement jump (CMJ) (mean value) to those played and not played matches in between.

	**Pre-match CMJ (cm)**	**Post-match CMJ (cm)**	**Post-match – Pre-match CMJ (cm) % |*p*| *d***
Matches in between	40.14 ± 3.54	39.06 ± 3.44	−3.02% **|**0.005**|** 0.43
No matches in between	41.19 ± 4.67	42.23 ± 4.86	2.32% **|**0.02**|** −0.21

**Table 3:** Description (mean ± SD) of accumulated training load and match load (ML) demands.

	**TD (m)**	**HSR (m)**	**HML (m)**	**ACC (*n*)**	**DCC (*n*)**	**Sprint (m)**	**CR-10 (A.U.)**	**sRPE (A.U.)**
ML	6,697.89 ± 2,550.21	457.77 ± 219.08	1,251.57 ± 500.62	64.33 ± 25.78	65.31 ± 26.55	40.55 ± 133.80	6.97 ± 1.34	500.68 ± 201.56
ATL	43,897.93 ± 10,747.09	2,453.60 ± 1,066.37	7,343.06 ± 1,870.24	550.15 ± 131.11	474.75 ± 131.11	75.56 ± 52.99	4.40 ± 0.69	3,152.51 ± 836.12

*ATL, Accumulated training load; ML, match load; TD, total distance; HSR, high-speed running; HML, high metabolic distance; ACC and DCC, high-intensity accelerations and decelerations (number of accelerations and decelerations at ≥ 3 m/s^−2^); CR-10, rate of perceived exertion at CR-10 Borg scale; sRPE, session-RPE (score at CR-10 multiplied by the duration of the session in minutes)*.

A two-way mixed design ANOVA with mean data of CMJ (Table 2) revealed a significant main effect of group condition, *F*_(1.74)_ = 5.06, *p* = 0.02, η^2^ = 0.06. The effect of time condition, *F* < 1, was not significant. The interaction between group and time condition revealed a significant effect, *F*_(1.74)_ = 10.14, *p* = 0.002, η^2^ = 0.12.

**Figure 2:**
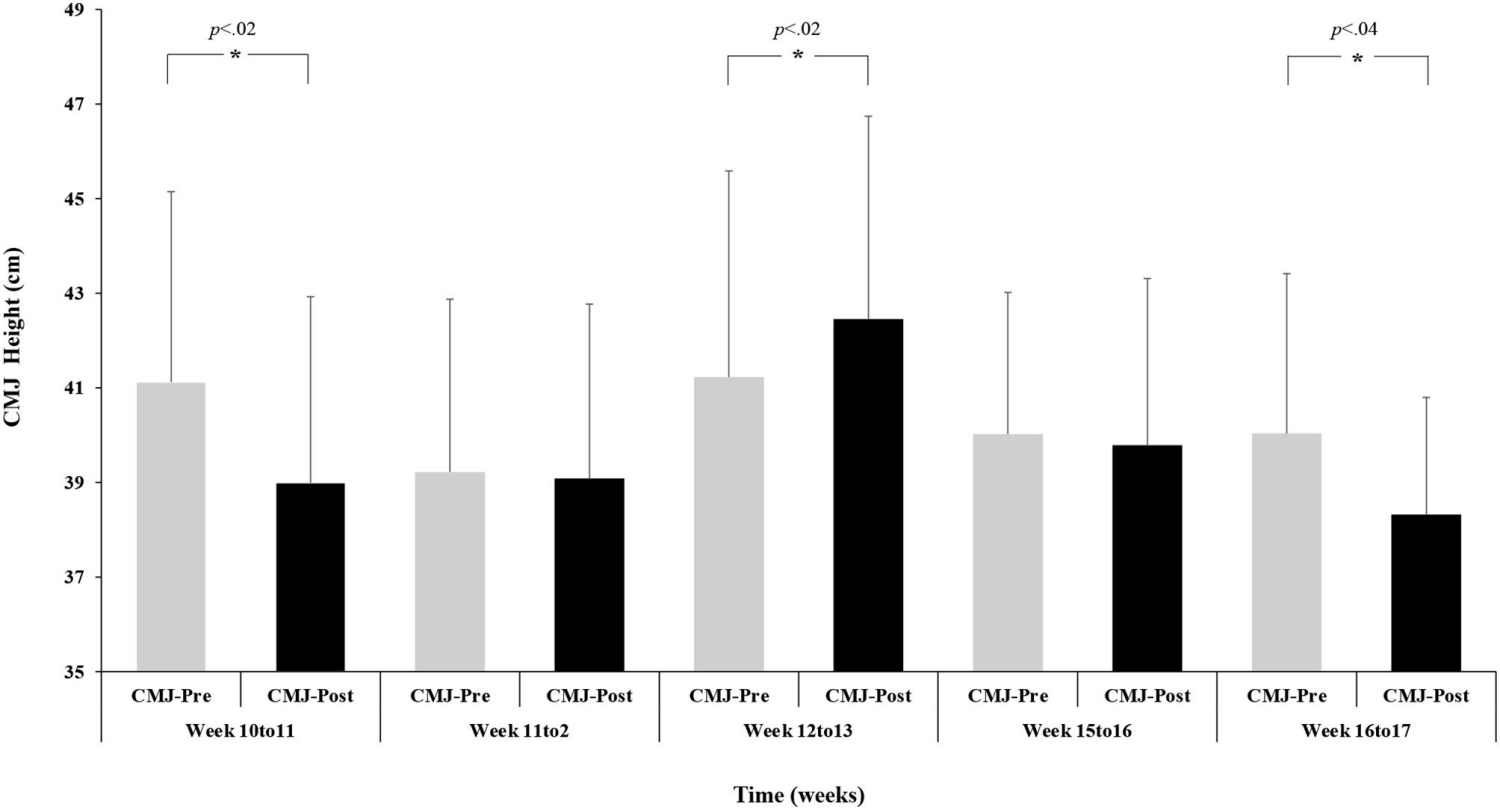
Pre- and post-match countermovement jump (CMJ) height (mean value ± SE) as a function of time (weeks).

A repeated-measures ANOVA with mean data of CMJ and time did not reveal any significant effect, *F*_(4.65)_ = 1.40, *p* = 0.24, η^2^ = 0.07, and *F*_(1.65)_ = 3.78, *p* = 0.056, η^2^ = 0.05, respectively. The interaction between group and time condition revealed a significant effect, *F*_(4.65)_ = 4.07, *p* = 0.005, η^2^ = 0.20. The pairwise comparisons showed significant differences between CMJ-pre and post in Week 10–11, 12–13, and 16–17, *t*_(15)_ = 2.50, *p* < 0.02, *d* = 0.53, *t*_(14)_ = 2.52, *p* < 0.02, *d* = 0.03, and *t*_(11)_ = 2.21, *p* < 0.04, *d* = – 0.28, respectively. A pairwise comparison between CMJ-pre and post in Week 11–12, Week 15–16 was not significant, *t*_(13)_ = 0.22, *p* < 0.82, *d* = 0.07, *t*_(12)_ = 0.39, *p* < 0.69, *d* = 58, respectively (refer to Figure 2 for more information).

At this point, we wondered about the possible associations between percentage of change of CMJ (pre-match – post-match) and the remaining variables (TD, HSR, HML, Sprint, ACC, DCC, CR-10, and sRPE) (refer to Table 3, for more information). Thus, the correlation analysis between the percentage of change of CMJ height and accumulated training load showed no significant correlations between percentage of change of CMJ height and HSR, Sprint, CR-10, and sRPE. However, negative moderate correlations were found between percentage of change of CMJ height and TD, HML, ACC and DCC (*r* = −0.38, *p* = 0.004; *r* = −0.33, *p* = 0.013; *r* = −0.39, *p* = 0.003, and *r* = −0.30, *p* = 0.026). A correlation analysis between the percentage of change of CMJ [CMJ (pre-match – post-match) and match load demands showed no significant correlations between any variable (refer to Table 4, for more information).

**Table 4:** Correlation between percentage of change of CMJ and accumulated training load and ML demands.

			**TD (m)**	**HSR (m)**	**HML (m)**	**ACC (*n*)**	**DCC (*n*)**	**Sprint (m)**	**CR-10 (A.U.)**	**sRPE (A.U.)**
ATL	% of change CMJ height	*r*	−0.3889	−0.1230	−0.3353	−0.3973	−0.3030	−0.1145	0.1169	−0.2314
		*p*	0.004^*^	0.37	0.013^*^	0.003^*^	0.026^*^	−0.41	0.40	0.092
ML	% of change CMJ height	*r*	0.0313	0.0287	0.0329	0.0663	0.0698	0.0653	0.0115	0.1136
		*p*	0.824	0.838	0.815	0.637	0.620	0.642	0.935	0.418

*ATL, Accumulated training load; ML, match load; TD, total distance; HSR, high-speed running; HML, high metabolic distance; ACC and DCC, high intensity accelerations and decelerations (number of accelerations and decelerations at ≥ 3 m/s^−2^); CR-10, rate of perceived exertion at CR-10 Borg scale; sRPE, session-RPE (score at CR-10 multiplied by duration of the session in minutes); CMJ, countermovement jump*.

* *refers to statistical significance at p <0.05*.

A multilinear regression analysis was performed to verify which variable of accumulated training load (agreement with the correlation analysis) could be used to better explain the percentage of change of CMJ Height. TD, HML, ACC and DCC were predictors of the percentage of change of CMJ Height (*r* = 0.27, *r* = 0.25, *r* = 0.31, and *r* = 0.22, respectively) (refer to Table 5, for more information).

**Table 5:** Values of regression analysis explaining the percentage of b^*^ change of CMJ b^*^ on the remaining R variables.

			b^*^	SE of b^*^	***R***	***R*^**2**^**	**Adjusted *R*^**2**^**	***F***	***p***
ATL	TD	% of ChangeCMJ Height	−0.27	0.13	0.27	0.07	0.05	4.14	0.04^*^
	HML	% of ChangeCMJ Height	−0.25	0.13	0.25	0.06	0.04	3.54	0.06
	ACC	% of ChangeCMJ Height	−0.31	0.13	0.31	0.09	0.07	5.58	0.02^*^
	DCC	% of ChangeCMJ Height	−0.22	0.13	0.22	0.05	0.03	2.89	0.09

*ATL, Accumulated training load; TD, total distance; HML, high metabolic distance; ACC and DCC, high-intensity accelerations and decelerations (number of accelerations and decelerations at ≥3 m s^−2^); CMJ, countermovement jump*.

* *refers to statistical significance at p <0.05*.

## Discussion

The purposes of this study were (1) to analyze variations in CMJ measures considering match participation and (2) to analyze the relationships between CMJ measures variations and ATL and ML values. The main findings revealed that match participation was the factor with the greatest influence on changes in both CMJ measures. The week without a match was the only one with significant increases in jump performance. The ATL values of TD, HML, ACC, and DCC had a greater influence (moderate correlations) than ML on changes in CMJ height. Also, TD, HML, ACC, and DCC predicted variations in both CMJ measures.

Regarding the first objective, the time condition (pre-post assessments) was not the main factor influencing differences in CMJ measures. Similar to these results, another study revealed that the time condition (pre- or post-assessments) had no significant effect on CMJ height changes (Stone et al., 2016). Indeed, a recent study that aimed to analyze variations in CMJ measures, including jump height, after a simulated soccer match revealed that none of the CMJ-related measures changed significantly between assessments (Lombard et al., 2020).

Notwithstanding the above-mentioned similarities, the above-mentioned studies were conducted on a small non-professional sample by using a simulated soccer match. Indeed, simulated soccer match protocols revealed similar physiological and technical demands as official matches (Russell et al., 2011). However, this type of protocol is usually tested over very short periods (pre- or post-assessments within a week). Other contextual factors, such as the quality of opposition and coach encouragement, might also influence the responses of the players during a training week and the official matches in-between (Guerrero-Calderón et al., 2021). Therefore, these different methodological approaches between studies make comparisons with these findings difficult.

Furthermore, in the present study, the only analyzed week sequence that showed increases in both CMJ measures was the weekly sequence without a match in between, namely, the period from Week 12 to Week 13. This appears to reinforce previous findings, revealing the fatigue effects that a single match has on jump performance (Stone et al., 2016; Lombard et al., 2020). This is especially true for CMJ performance due to its natural characteristics (SSC abilities), suggesting a need for longer (>72 h) recovery periods (Silva et al., 2018). This highlights the need for practitioners to analyze CMJ height for up to 72 h after a match to ensure that athletes who participated in the match were given enough time to recover and are ready to train. By doing this, coaches can be more confident in their decisions to select specific players for the next match.

Interestingly, these results revealed that the ATL of all the analyzed accelerometry-based GPS metrics (HML, ACC, and DCC) had moderate relationships with the percentage of change of CMJ height. At the same time, no associations were found for ML. Another study that assessed neuromuscular performance before and after a youth soccer match revealed that the isometric strength of knee extensors and flexors and the rate of force development decreased by up to ~10%, whereas CMJ performance remained unchanged (Thorlund et al., 2009). This is somewhat similar to these results, as the authors of the aforementioned study considered only pre- or post-match (ML) CMJ changes without considering the preceding weekly training loads (ATL) as in the present study (Thorlund et al., 2009).

However, another study revealed that CMJ height was impacted by match load but only between 0.5 and 18 h post-match (Rowell et al., 2017). The authors of the above-mentioned study suggested that CMJ height is more sensitive to track immediate neuromuscular changes after a match, while the FC:CT ratio seems better suited to track neuromuscular changes at longer time-frames (Rowell et al., 2017). This fact might explain why there were no relationships between the percentage of change of CMJ measures and match load demands in the present study.

Furthermore, these findings showed no significant relationships between the percentage of change of CMJ measures and ATL and ML subjective internal load measures. A previous study conducted on 19 professional soccer players revealed that sRPE (relative to leg muscle effort perceptions) and its related accumulated training load had negative correlations (*r* range = −0.52 to −0.61) with changes in CMJ height after a 9 week training period with matches in between (Arcos et al., 2015). However, it must be highlighted that the negative correlations found in that study were related to changes in single-leg (dominant- and non-dominant) CMJ protocols (Arcos et al., 2015). Indeed, the above-mentioned study revealed no significant associations between sRPE measures and bilateral CMJ changes (*r* range = −0.17 to −0.20), using the both-hands-on-hips protocol (as in the present study), which is in concordance with these findings. To the best of our knowledge, no other study has tested these relationships. Thus, future studies should confirm this lack of relationships.

It was previously documented that soccer match demands are associated with acute and residual fatigue effects on the performance of soccer players (Nedelec et al., 2011; Silva et al., 2013). As revealed in the present study, it seems that the ATL of accelerometry-based metrics and TD might have a greater impact, at least on the neuromuscular fatigue of players. Indeed, the multilinear regression confirmed that the weekly training loads of TD, HML, ACC, and DCC were the best predictors of the percentage changes in CMJ performance.

Interestingly, a study conducted on 27 professional soccer players revealed that weekly TD and the number of high accelerations/decelerations are three to four times greater than the demands of a soccer match (Clemente et al., 2019). Based on such findings, it can be argued that the ATL of these metrics has a stronger fatigue effect than only ML on neuromuscular performance (as demonstrated in the present study). That is, the players with higher ATL of TD, HML, ACC, and DCC are less likely than other players to experience improved jump performance, 72 h post-match. However, the short duration maximal CMJ jump test was conducted 1 day after the first weekly training session (MD-4; recovery day) in the present study, which might have influenced such findings.

Although, coaches should exercise some caution when planning weekly high accelerations and decelerations (≥3 m s^−2^) ATL, as they have been associated with high neuromuscular fatigue levels and increased injury risk if not monitored properly (Harper et al., 2019). Indeed, a study conducted on 15 elite U-19 soccer players revealed correlations between decelerations (>2 m s^−2^) and CMJ concentric and eccentric forces (de Hoyo et al., 2016). The above-mentioned study also suggested that accelerometry-based metrics can predict neuromuscular performance changes up to 48 h after a match (de Hoyo et al., 2016). For those reasons, the negative effects that accelerometry-based metrics have on neuromuscular performance, especially deceleration measures, must be addressed (Gastin et al., 2019). Also, coaches and practitioners must carefully manage these ATL-related metrics and ensure that the appropriate strategies are followed to increase the resilience of players to deceleration and acceleration loads (Harper and Kiely, 2018).

This study had some limitations. One of the main limitations was the small sample size. However, this is a common issue in studies conducted in professional team settings. Furthermore, the use of a short-duration maximal jump test protocol during MD-4 (recovery) training sessions (72 h after the previous match) could have influenced the results. Future studies should analyze variations in CMJ measures before the start of a new training microcycle (e.g., on MD-5). Another limitation is related to the CMJ measures used, as they may not be the most sensitive to the assessed changes. However, a force platform was not available to implement other measures. Future studies should use other neuromuscular measures to reinforce the present findings.

## Conclusion

Match participation was the main factor influencing CMJ performance. The ATL values of HML, ACC, DCC, and TD had moderate correlations with both percentage changes of CMJ measures. Those metrics were also the best predictors of the percentage changes of CMJ measures according to linear regression analysis. Coaches and practitioners must monitor and manage weekly accelerometry-based metrics considering the next match and implement strategies to increase the resilience of players to accelerometry demands.

## Data Availability Statement

The raw data supporting the conclusions of this article will be made available by the authors, without undue reservation.

## Ethics Statement

The studies involving human participants were reviewed and approved by Escola Superior de Desporto e Lazer, Instituto Politécnico de Viana do Castelo. The patients/participants provided their written informed consent to participate in this study.

## Author Contributions

RS and FC lead the project, established the protocol, and written, and revised the original manuscript. FG-F and LPA have also written and revised the original manuscript. AB helped in data collection and revised the final document. All authors contributed to the article and approved the submitted version.

## Conflict of Interest

The authors declare that the research was conducted in the absence of any commercial or financial relationships that could be construed as a potential conflict of interest.

## Publisher's Note

All claims expressed in this article are solely those of the authors and do not necessarily represent those of their affiliated organizations, or those of the publisher, the editors and the reviewers. Any product that may be evaluated in this article, or claim that may be made by its manufacturer, is not guaranteed or endorsed by the publisher.
